# Optimizing the Size of Zr-Based Metal–Organic Frameworks for Enhanced Anticancer Efficacy

**DOI:** 10.3390/nano15110826

**Published:** 2025-05-29

**Authors:** Zan Cheng, Mei Yu, Yilong Wan, Huandong Xiang, Haoran Wei, Xu Zu, Xin Li, Ruiting Zhang, Fangshu Li, Shanshan Wang, Yongxin She

**Affiliations:** 1 Institute of Quality Standards & Testing Technology for Agro-Products, Chinese Academy of Agricultural Sciences, Beijing 100081, China; 82101222151@caas.cn (Z.C.);; 2 CAS Key Laboratory for Biomedical Effects of Nanomaterials and Nanosafety, Institute of High Energy Physics and National Center for Nanoscience and Technology, Chinese Academy of Sciences, Beijing 100049, China

**Keywords:** metal–organic frameworks, UiO-67, drug delivery, cellular internalization, anticancer

## Abstract

Metal–organic frameworks (MOFs) have great potential for drug delivery systems due to their tunnel pore size, structural versatility, and high surface area. Among them, UiO-67 have recently attracted substantial attention as functional nanocarriers for effective delivery of small molecule chemical drugs. However, the influence of the size on cellular uptake of UiO-67 remains ambiguous. Here, we use polyvinyl pyrrolidone (PVP) as the capping agent of UiO-67 to synthesize spherical Zr-based MOFs with various diameters, including 40 nm, 60 nm, and 120 nm. The highest cellular uptake is observed in the case of Zr-based MOFs with a diameter of 40 nm (PU_40_ MOFs). Moreover, doxorubicin can be loaded into the inner pores of PU_40_ MOF via π-π and electrostatic interactions (DPU_40_ MOFs), with a loading capacity of 82 wt%, and gradually released under acidic conditions. In vitro, the resulting DPU_40_ MOFs can be internalized by cancer cells more effectively, thereby enhancing the delivery of doxorubicin into cancer cells. Ultimately, this results in enhanced antitumor efficacy toward 4T1, Hs 578T, and MCF-7 cells. Our findings indicate that approximately 40 nm may be the optimum diameter for the special Zr-based MOFs to be internalized by cells more effectively, providing potent potential nanocarriers for drug delivery.

## 1. Introduction

As clearly demonstrated by previous studies, nanomaterials, such as graphene [1,2], mesoporous SiO_2_ [3,4], and polymeric nanoparticles [5,6], can be used as functional nanocarriers for drug delivery, enhancing the therapeutic efficacy of chemical drugs while minimizing their side effects toward normal cells via improving their water solubility and bioavailability, or even controlling their release at the tumor sites. These positive outcomes mainly result from the features of nanocarriers, including size, surface charge, and morphology, which play significant roles in navigating various biological barriers such as cellular membranes [7,8,9]. So far, some efforts have been devoted to investigating the influence of the size of nanocarriers on their cellular internalization, which is the first step of the delivery process. For example, nanocarriers smaller than 10 nm can easily penetrate into the cell [10]. As the size further increases, nanocarriers are internalized through active processes, including caveolae-based endocytosis [11], clathrin-mediated endocytosis [12], phagocytosis [13], and micropinocytosis [14]. Notably, some reports have demonstrated that nanocarriers with a size of approximately 50 nm can be internalized by cells more efficiently and have a higher uptake rate [15,16]. In vivo, the biodistribution and blood circulation time of nanocarriers are also dependent on their size. Nanocarriers with sizes ranging from 40 to 100 nm can avoid the scavenging effect of reticuloendothelial systems more effectively [17,18].

Recently, metal–organic frameworks (MOFs) are emerging nanocarriers for the delivery of chemical drugs due to their high surface area, tunable pore size, and structural versatility [19,20,21]. For example, zinc-based, iron-based, and copper-based MOFs exhibit great advantages in delivering low molecular weight chemical drugs into cancer cells, causing enhanced killing effects on A549 [22], MDA-MB-231 [23], and 4T1 cells [24]. Among them, zirconium (Zr)-based MOFs, with rather chemical stability toward moisture and acids, as well as good biocompatibility, have drawn increasing attention toward the effective delivery of small molecule chemical drugs [25,26,27]. For instance, UiO-66 has been used to load quercetin, enhancing its accumulated amount in the tumor to reduce the dosage used [28]. Moreover, Zr-based MOFs are easily modified by functional moieties via the chemical reaction with carboxyl and hydroxyl groups localized on their surface or coordination interaction with zirconium, conferring them with desired biological features, such as targeting ability [29]. For instance, phosphate-terminal DNA aptamer-conjugated Zr-based porphyrinic MOF nanoparticles were fabricated through strong coordination between zirconium and phosphate, which enabled target-specific bioimaging and improved the photodynamic therapy effect [30]. In particular, UiO-67 MOFs use biphenyl dicarboxylic acid and its derivatives as organic ligands to link with Zr^4+^ ions and thus exhibit larger pore channels compared to UiO-66 MOFs made of terephthalic acid and its derivatives. This makes UiO-67 MOFs rather suitable to load chemical drugs with various sizes [31,32]. Considering that the mechanism of the interaction between UiO-67 MOFs and cancer cells is still ambiguous, it is necessary to understand the influence of the size on their uptake, finally helping guide the design of highly efficient nanocarriers for drug delivery. To address this issue, the main difficulty is fine-tuning the size of UiO-67 MOFs while simultaneously showing remarkable drug loading efficacy as well as good biocompatibility.

**Scheme 1:**
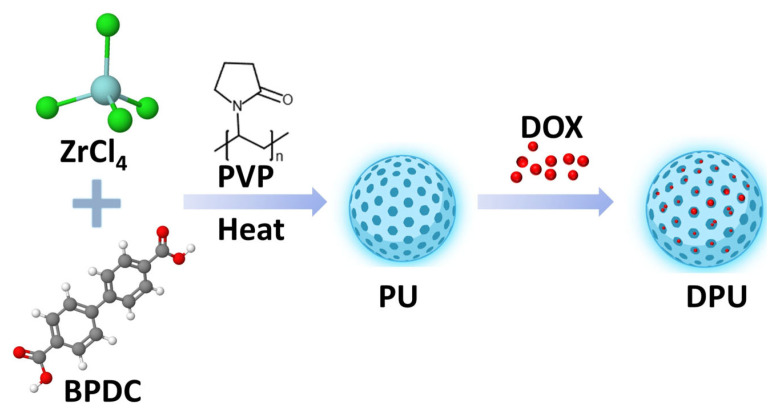
Schematic illustration of the preparation of PU_x_ MOFs and their applications for DOX loading.

In this work, based on the unique steric property of polyvinylpyrrolidone (PVP) [33,34,35], we use it as the capping agent of UiO-67 to prepare spherical Zr-based MOFs (Scheme 1). The physical and chemical properties of the prepared nanomaterials are characterized by scanning electron microscopy (SEM), transmission electron microscopy (TEM), dynamic light scattering (DLS), UV–Vis spectroscopy, Brunauer–Emmett–Teller (BET) surface area analysis, powder X-ray diffraction (pXRD), and X-ray photoelectron spectroscopy (XPS). By adjusting the reaction temperature, Zr-based MOFs with various diameters, including 40 nm, 60 nm, and 120 nm, are prepared through the solvothermal method and further labeled by fluorescein isothiocyanate (FITC) to trace their cellular uptake process. Furthermore, we choose the as-prepared Zr-based MOFs with a diameter of 40 nm (PU_40_) as the nanocarrier to load the chemical agent DOX and then preferentially deliver it within cancer cells, resulting in an enhanced killing effect compared with free DOX. These results demonstrate that 40 nm Zr-based MOFs show much higher internalization efficiency compared to 60 nm and 120 nm counterparts and provide an alternative platform for drug delivery.

## 2. Materials and Methods

**Materials.** Zirconium tetrachloride (ZrCl_4_, 99.9%) and PVP (M.W. 58,000, ≥99.0%) were purchased from Shanghai Aladdin Biochemical Technology Co., Ltd. (Shanghai, China). Biphenyl-4,4′-dicarboxylic acid (BPDC, 97%) and doxorubicin hydrochloride (DOX, 98%) were obtained from Shanghai Yuanye Bio-Technology Co., Ltd. (Shanghai, China). Dimethyl Sulfoxide (DMSO, 99.9%) and N, N-Dimethylformamide (DMF, 99.9%) were purchased from Beijing InnoChem Science & Technology Co., Ltd. (Beijing, China). Fluorescein isothiocyanate (FITC, ≥90%) was purchased from Beijing Solarbio Science & Technology Co., Ltd. (Beijing, China). Penicillin and streptomycin (1000 U mL^−1^) were purchased from Thermo Fisher Scientific Inc. (Waltham, MA, USA). Other chemicals were purchased from Beijing Chemical Reagent Co., Ltd. (Beijing, China). All chemical reagents were used as received without further purification. The 4T1, MCF-7, and Hs 578T cell lines were provided by Wuhan Pricella Biotechnology Co., Ltd. (Wuhan, China).

**Synthesis of Zr-based MOFs.** According to the modified solvothermal method [36,37], 26.8 mg of ZrCl_4_ and 100 mg of PVP were added into 15 mL of DMF under ultrasonication for 10 min, finally obtaining dispersion A. Meanwhile, 35 mg of BPDC was added to another 10 mL of DMF and ultrasonicated for 10 min, obtaining solution B. Afterwards, solution B was slowly dropped into dispersion A under ultrasonication, and the resultant mixture was transferred into a Teflon-lined stainless steel autoclave to be heated at 90 °C, 120 °C, or 150 °C for 10 h. When naturally cooling to room temperature, the precipitate was collected by centrifugation and washed twice with DMF and ethanol to remove residual solvents and byproducts (designated as PU_x_ MOFs, x = 40, 60, and 120).

**Synthesis of FITC-labeled Zr-based MOFs.** In brief, 0.5 mL of DMSO solution containing FITC was added to 0.5 mL of PU_x_ dispersion in DMSO. After ultrasonication for 5 min, the resultant mixture was shaken in the dark for 24 h. Finally, the precipitates were collected by centrifugation and washed three times with deionized water (designated as FPU_x_ MOFs, x = 40, 60, and 120).

**Physical and chemical characterization.** The morphology and size in the dry state of the products were measured using field-emission scanning electron microscopy (FE-SEM, SU-8100, Hitachi, Ltd., Tokyo, Japan) at an acceleration voltage of 3 keV. A transmission electron microscopy (TEM, Tecnai G2 F20 S-TWIN, 200 kV, FEI, Hillsboro, OR, USA) coupled with energy-dispersive X-ray spectroscopy (EDS) micro-analysis was used to characterize the microstructure and elemental distribution of the nanomaterials. Powder X-ray diffraction (pXRD) patterns were recorded using a Bruker D8 Advance diffractometer with Cu Kα radiation (Bruker, Karlsruhe, Germany) to confirm the crystalline structure of the synthesized nanomaterials. Dynamic light scattering (DLS, Malvern Panalytical, Malvern UK) was used to determine the hydrodynamic diameter and zeta potential of the samples to evaluate their dispersion stability and surface charge in aqueous environments, which are critical for biological applications. Ultraviolet-visible (UV-Vis) spectra were obtained using a U-3900 spectrophotometer (Hitachi, Ltd., Tokyo, Japan) to analyze the optical absorption properties of DOX, PU_40_ MOFs, and PU_40_ MOFs loaded with DOX, which could be used to confirm the successful loading of DOX into PU_40_ and calculate the loading capability. X-ray photoelectron spectroscopy (XPS) analysis was conducted using a Thermo Escalab 250XI spectrometer (Thermo Fisher Scientific, USA) to investigate the elemental composition and the chemical states of the prepared materials, providing insights into the presence of functional groups. The specific surface area and pore size distribution of the samples were determined using a Micromeritics ASAP 2460 analyzer (Micromeritics, Norcross, GA, USA). The specific surface area was calculated using the Brunauer–Emmett–Teller (BET) method, and the pore size distribution was obtained based on the Barrett–Joyner–Halenda (BJH) method.

**Evaluation of cellular uptake.** 4T1 cells were incubated with Dulbecco’s Modified Eagle’s Medium (DMEM, Corning Inc., Corning, NY, USA) supplemented with 10% fetal bovine serum (FBS, Corning), 200 U mL^−1^ of penicillin, and 200 U mL^−1^ of streptomycin at 37 °C in a humidified 5% CO_2_ atmosphere. First, 4T1 cells were seeded into 24-well plates at a density of 50,000 cells per well. After incubation for 24 h, the culture media were replaced with fresh complete media containing PU_x_ MOFs (10 μg mL^−1^). At given time points (4, 8, 12, and 24 h), these treated cells were collected and digested with 200 μL concentrated nitric acid and diluted to 5 mL. The obtained solutions were used to quantify the concentration of Zr^4+^ ions via inductively coupled plasma mass spectrometry analysis (ICP-MS, Thermo Elemental X7, Thermo Fisher Scientific, Waltham, MA, USA).

Alternatively, 4T1 cells were seeded onto the confocal dishes at a density of 50,000 cells per well and incubated for 24 h. The culture media were then replaced with complete media containing FPU_x_ MOFs (10 μg mL^−1^). At given time points (4, 8, 12, and 24 h), these treated 4T1 cells were stained with 4′,6-Diamidino-2-Phenylindole (DAPI, Becton Dickinson, Franklin Lakes, NJ, USA) for 10 min and subsequently washed three times with phosphate-buffered saline (PBS, pH 7.40, Gibco, Thermo Fisher Scientific). Finally, confocal laser-scanning fluorescence images were recorded using laser confocal fluorescence microscopy (Nikon A1R-si, Nikon, Shinjuku, Japan) to visualize the cellular uptake and intracellular localization of FPU_x_ MOFs, thereby evaluating their cellular internalization efficiency.

**Loading of doxorubicin into PU_40_ MOFs.** Typically, 0.5 mL of DOX aqueous solution was added into 0.5 mL of PU_40_ MOFs aqueous dispersion. After incubation overnight under ambient conditions, the resultant mixture was centrifuged at 12,000 rpm for 10 min and then washed three times to remove free DOX. Meanwhile, the supernatant was collected to quantify the concentration of free DOX by measuring the absorbance at 480 nm using a UV-vis spectrophotometer. The drug loading capability (DLC) of PU_40_ MOFs was calculated according to the following equation [38,39]:

(1) $$\mathrm{DLC}\;(\%)=\frac{\mathrm{Mass}\;\mathrm{of}\;\mathrm{DOX}\;\mathrm{fed}-\mathrm{Mass}\;\mathrm{of}\;\mathrm{DOX}\;\mathrm{in}\;\mathrm{the}\;\mathrm{supernatant}}{\mathrm{Mass}\;\mathrm{of}\;{\mathrm{DPU}}_{40}\;MOFs}\times 100,$$

**Release of doxorubicin from Zr-based MOFs.** In brief, 8 mg of DPU_40_ MOFs was dispersed into 16 mL of PBS (pH 5.50 mimicking the pH of acidic intracellular compartments and pH 7.40 mimicking physiological pH) and incubated at 37 °C [40]. At specific intervals, 1 mL of the dispersion was taken out and centrifuged at 12,000 rpm for 10 min. Finally, the supernatant was collected for UV-vis analysis at the absorbance of 480 nm [41].

**Cellular toxicity assay.** Typically, 4T1, human triple-negative breast cancer (Hs 578T), and human breast cancer cells (MCF-7) were seeded into 96-well plates at a density of 8000 cells per well and incubated for 24 h. PU_40_ MOFs, DPU_40_ MOFs, or free DOX were added at their specified concentrations and incubated for another 24 h. The culture media were replaced with 100 μL of cell counting kit-8 (CCK-8, Dojindo Lab., Kumamoto, Japan) solution and these treated cells were incubated for 2 h at 37 °C. Finally, the absorbance at 450 nm was measured using a Thermo Fisher Multiskan MK3 microplate reader (Thermo Fisher Scientific, Waltham, MA, USA).

## 3. Results

**Figure 1:**
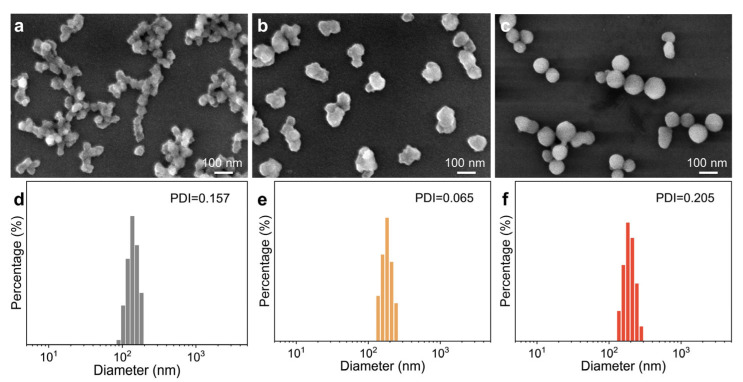
SEM images and hydrodynamic diameters of Zr-based MOFs synthesized at 90 °C (**a**,**d**), 120 °C (**b**,**e**), and 150 °C (**c**,**f**).

As known, reaction temperature plays an important role in controlling the size of nanoparticles by affecting the energy available for crystalline growth [42,43,44]. During the solvothermal process, Zr^4+^ ions can quickly coordinate with BPDC to form primary particles. Meanwhile, PVP acts as the capped agent that adsorbs onto the surface of these primary particles to affect their growth process [45,46,47,48], ultimately leading to the formation of spherical PVP-coated Zr-based MOFs. Thus, we try to change the reaction temperature to fine-tune the diameter of Zr-based MOFs in the presence of PVP. As shown in Figure 1a, at the reaction temperature of 90 °C, the synthesized Zr-based MOFs are indeed spherical and show an average diameter of approximately 40 nm obtained by SEM. With the increase in the reaction temperature, the diameter of Zr-based MOFs grows to ~60 nm at 120 °C (Figure 1b), and further to ~120 nm at 150 °C (Figure 1c). Notably, the absence of PVP results in distinct changes in morphology and size of Zr-based MOFs, indicative of PVP playing a significant role in controlling their morphology features (Figure S1). Moreover, DLS data acquired in aqueous dispersions indicate that the hydrodynamic diameters of Zr-based MOFs are, respectively, 100 nm when synthesized at 90 °C (Figure 1d), 180 nm when synthesized at 120 °C (Figure 1e), and 280 nm when synthesized at 150 °C (Figure 1f), accompanied by a polydispersity index (PDI) value of ~0.200. These demonstrate that the as-synthesized Zr-based MOFs show narrow size distribution. Notably, the particle size of the samples was characterized using both scanning electron microscopy (SEM) and dynamic light scattering (DLS). SEM measurement reflects the physical diameter of dried particles under vacuum, while DLS provides the hydrodynamic diameter in aqueous suspension, which tends to be larger due to the surrounding solvation layer. In this study, the physical diameter obtained from SEM was primarily used throughout the manuscript to represent particle size, as it better reflects the core structure and morphology of the prepared MOFs.

**Figure 2:**
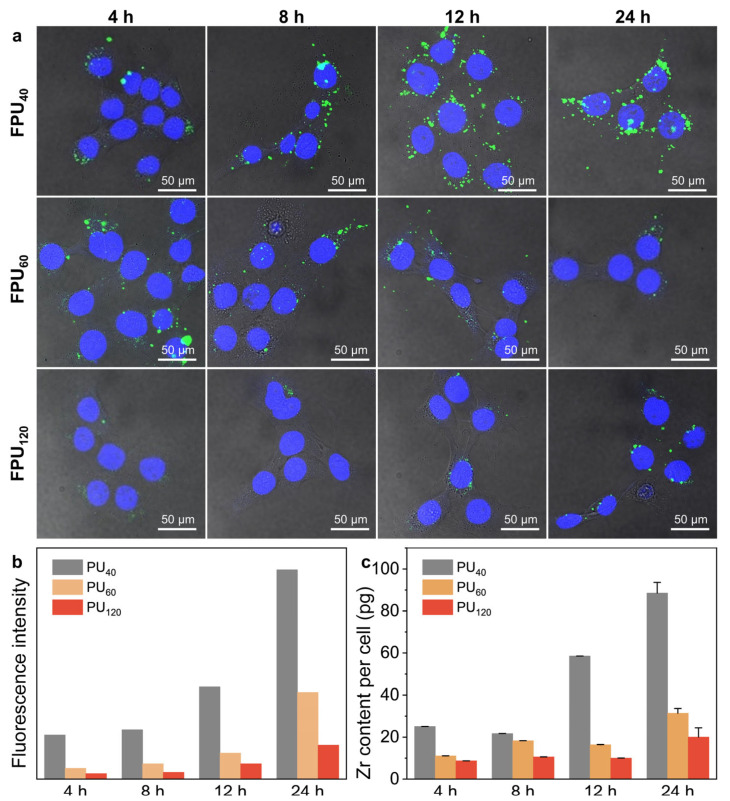
(**a**) Confocal fluorescence images of 4T1 cells incubated with various Zr-based MOFs. (**b**) Corresponding fluorescence intensity of the confocal fluorescence images. (**c**) Quantitative analysis of Zr^4+^ ion within cells.

Next, we investigated the influence of the diameter of Zr-based MOFs on their cellular internalization. As shown in Figure 2a, for Zr-based MOFs with a diameter of 40 nm, it is clear that the green fluorescence of FITC within 4T1 cells becomes brighter over time, indicating that the uptake of PU_40_ MOFs is time-dependent, in accordance with the changes in the cellular concentration of Zr^4+^ ions. When the diameter of UiOs increases to 60 nm, the fluorescence intensity of FITC also increases over incubation time but is obviously lower than that in the case of PU_40_ MOFs (Figure 2b). With a diameter up to 120 nm, almost no green fluorescence can be observed until the 4T1 cells are incubated for more than 8 h. Taken together with the results of ICP-MS analysis (Figure 2c), it is inferred that the cellular internalization of Zr-based MOFs exhibits a size-dependent manner and the 40 nm diameter would permit maximal rate of intracellular concentration within 4T1 cells.

**Figure 3:**
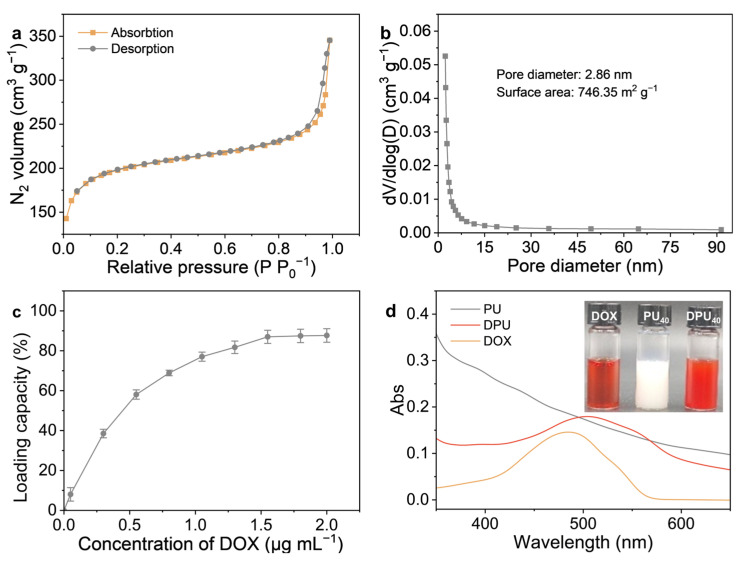
(**a**) Nitrogen gas adsorption–desorption isotherms of PU_40_ MOFs. (**b**) Pore size distribution of PU_40_ MOFs. (**c**) Loading capacity of DPU_40_ MOFs upon various DOX input concentrations. (**d**) Ultraviolet-visible spectrum of PU_40_ MOFs, DPU_40_ MOFs, DOX, and photo images of DOX, PU_40_ MOFs, and DPU_40_ MOFs in aqueous solution.

Furthermore, we chose PU_40_ MOFs as the nanocarriers to deliver DOX. The pore diameter and specific surface area of PU_40_ MOFs were determined using Barrett–Joyner–Halenda and Brunauer–Emmett–Teller methods, respectively (Figure 3a). N_2_ adsorption–desorption isotherm demonstrates that PU_40_ MOFs show an average pore diameter of 2.86 nm, a micropore volume of 0.34 cm^3^ g^−1^, and a specific surface area of 746.35 m^2^ g^−1^ (Figure 3b). This reveals the porous structure of PU_40_ MOFs, which is further demonstrated by TEM images (Figure S2). Benefiting from the high degree of porosity and extensive surface area, PU_40_ MOFs can absorb DOX within their inner pores, with a loading capacity of approximately 82 wt% (Figure 3c). Remarkably, as shown in Figure 3d, the formation of DPU_40_ MOFs is very visible as the color of the PU_40_ MOFs’ aqueous dispersion varies from white to reddish. According to the UV-vis spectra, the characteristic absorbance peak of DOX at ~480 nm superimposes on the absorption spectrum of PU_40_ MOFs, and a red shift of 20 nm is observed clearly. These undoubtedly indicate the successful loading of DOX into the inner pores of PU_40_ MOFs largely via simple π-π and electrostatic interactions between DOX and BPDC, similar to those with carbon-based nanomaterials, such as graphene [49,50,51] and carbon nanotubes [52,53].

**Figure 4:**
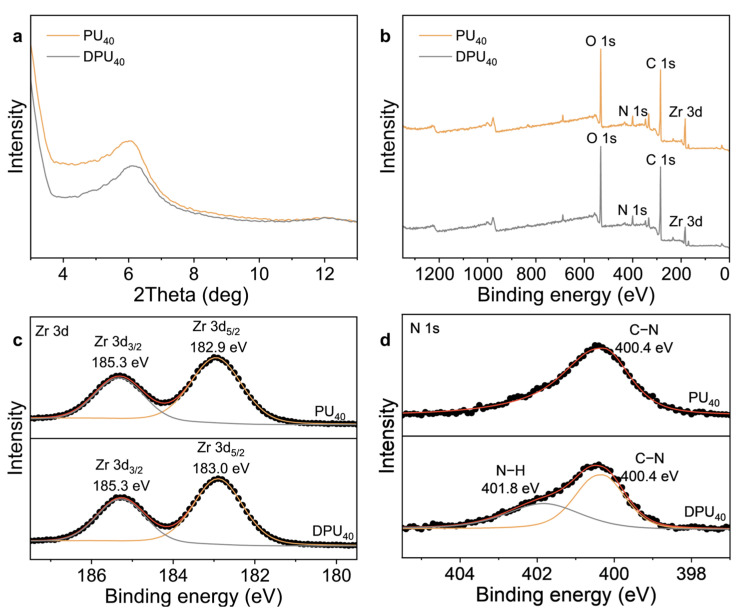
(**a**) Powder X-ray diffraction of PU_40_ MOFs and DPU_40_ MOFs. (**b**) Survey X-ray photoelectron spectroscopy of PU_40_ MOFs and DPU_40_ MOFs. X-ray photoelectron spectroscopy spectrum in the region of Zr 3d (**c**) and N 1s (**d**) of PU_40_ MOFs and DPU_40_ MOFs.

The pXRD pattern of DPU_40_ MOFs shows the characteristic peak of PU_40_ MOFs, indicating that there is no significant change in the crystal structure. However, a shift of ~0.2 ° is observed after loading DOX (Figure 4a). This indicates that DOX molecules are capped in the inner pore of PU_40_ MOFs via strong π-π and electrostatic interactions between each other [54]. The survey XPS spectra of PU_40_ MOFs and DPU_40_ MOFs were also performed. Note that among these spectra, three peaks at the binding energies of 184, 400, and 285 eV can be attributed to Zr, N, and C elements [55] (Figure 4b). This is in accordance with the energy-dispersive X-ray spectroscopy (EDS) mapping (Figure S2b) and verifies the presence of Zr, N, and C elements in the prepared materials, indicative of the successful preparation of PU_40_ MOFs. The deconvolution of the Zr 3d XPS spectrum in PU_40_ MOFs is well fitted with two peaks, 185.3 and 182.9 eV, which are assigned to Zr 3d_3/2_ and Zr 3d_5/2_, respectively [56,57] (Figure 4c). For DPU_40_ MOFs, the binding energy of 182.9 eV shifts toward a higher value of 183.0 eV. These findings suggest that there is electron transfer between DOX and PU_40_ MOFs. Moreover, a new peak at 401.8 eV, corresponding to the N-H bond [58], emerges in the N 1s XPS spectrum of PU_40_ MOFs (Figure 4d), further demonstrating the successful loading of DOX into their inner pores.

**Figure 5:**
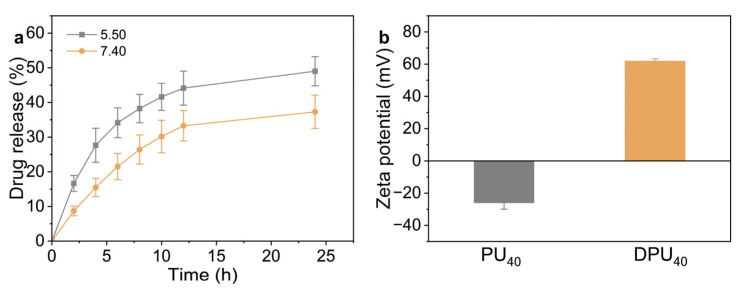
(**a**) Drug release from DPU_40_ MOFs in PB (pH 5.50 or 7.40). (**b**) Zeta potential of PU_40_ MOFs and DPU_40_ MOFs.

Figure 5a shows the release profiles of PU_40_ MOFs in PBS buffers with different pH values (7.40 and 5.50) at 37 °C. It is worth noting that the burst effect cannot be detected, demonstrating that the loaded DOX mostly distributes within the inner channels and cavities of PU_40_ MOFs. Remarkably, about 34% of DOX is released at pH 7.40 in 12 h, while it takes only 5 h to release the same amount of DOX at pH 5.50. This confirms that the release rate of DOX from PU_40_ MOFs is much faster in the acidic media, showing a pH-responsive manner. Considering that the Zeta potential elevates from −27 mV to 60 mV (Figure 5b), the pH-responsive release profiles of PU_40_ MOFs are largely due to the partial protonation of amino groups in DOX in the acidic condition [59,60], which can weaken the interaction between DOX and PU_40_ MOFs.

**Figure 6:**
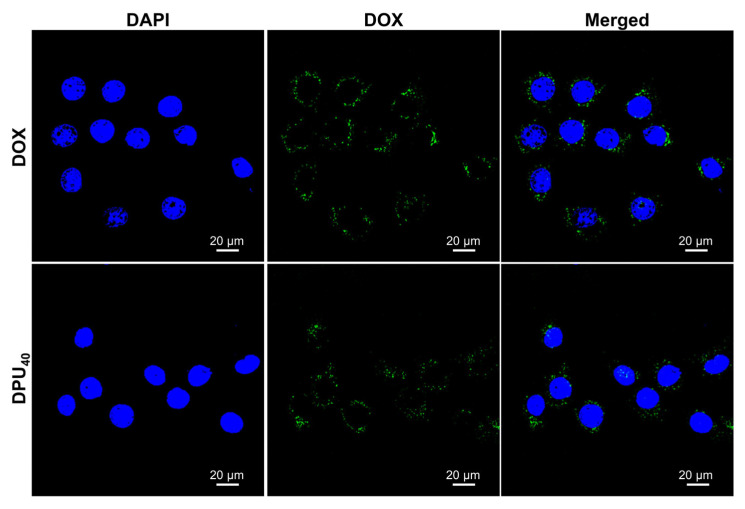
Confocal laser-scanning fluorescence images of 4T1 cells treated with DOX and DPU_40_ MOFs.

Motivated by the enhanced cellular uptake and pH-responsive release profile, we continued to examine the efficacy of DPU_40_ MOFs for chemotherapy in vitro. Prior to using PU_40_ MOFs for biomedical applications, their biocompatibility was evaluated on 4T1 cells using CCK-8 assay. Figure S3 shows that no obvious cytotoxicity is observed after incubation with PU_40_ MOFs at different concentrations for 24 h, indicating their good biocompatibility and the potential to be used as functional nanocarriers for enhanced delivery of DOX within cancer cells. Then, the internalization behavior of DPU_40_ MOFs in 4T1 cells was observed using confocal laser scanning microscopy. As depicted in Figure 6, green fluorescence of DOX can be detected in the nuclei and cytoplasmic regions of these 4T1 cells treated with DPU_40_ MOFs. More importantly, these 4T1 cells treated with DPU_40_ MOFs exhibit much stronger fluorescence intensity than those 4T1 cells treated with free DOX. This demonstrates the efficient cellular uptake of DPU_40_ MOFs for effective delivery of DOX.

**Figure 7:**
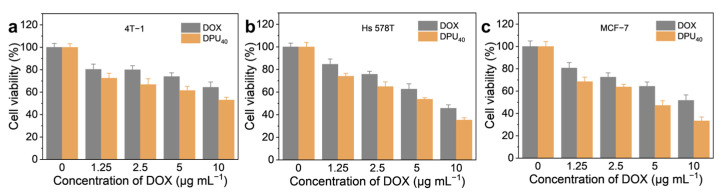
Cytotoxicity of DOX and DPU_40_ MOFs on (**a**) 4T1, (**b**) Hs578T, and (**c**) MCF-7.

Finally, the in vitro cell viability of free DOX and DPU_40_ MOFs at various concentrations on 4T1 cells was evaluated using the CCK-8 assay. Significantly, as shown in Figure 7a, free DOX shows an incremental inhibitory effect against 4T1 cells with the increase in the concentration of DOX, and about 37% of 4T1 cells are killed under 10 μg mL^−1^ of DOX. However, due to the faster release of loaded DOX inside the endosomal compartment of 4T1 cells induced by low pH, higher cytotoxicity of DPU_40_ MOFs is observed compared to free DOX at the same equivalent DOX concentration. Encouragingly, 2.5 μg mL^−1^ of loaded DOX causes to kill 35% of 4T1 cells, while 10 μg mL^−1^ of free DOX is required to achieve the same killing effect. Similarly, similar trends are also observed in both Hs 578T and MCF-7 cells (Figure 7b,c). These results demonstrate that PU_40_ MOFs are safe and effective nanocarriers capable of delivering chemical drugs into cancer cells to enhance killing efficacy.

## 4. Conclusions

In summary, we use PVP as a capped agent to successfully synthesize spherical Zr-based MOFs with different diameters via adjusting reaction temperature. The results of cellular uptake assays demonstrate that the diameter of Zr-based MOFs has a great influence on their internalization and the 40 nm diameter is desired to reach a maximal rate of intracellular concentration. Moreover, due to outstanding high porosity, 40 nm Zr-based MOFs can successfully load DOX into their inner pores with a drug loading of approximately 82 wt%. After effective uptake of DPU_40_ MOFs by cancer cells via receptor-mediated endocytosis, the loaded DOX can be sustainedly released at a faster rate in the acidic media and results in an enhanced killing effect toward cancer cells compared to free DOX, exhibiting superior anti-tumor efficacy and triggering cell death in multiple cancer cell lines, including MCF-7, Hs 578T, and 4T1 cells. These findings highlight the therapeutic advantages of the MOF-based delivery system and support its potential application in targeted cancer therapy. In combination with their good biocompatibility, Zr-based MOFs with a diameter of 40 nm are suitable to be used as functional nanocarriers for drug delivery. Moreover, this work provides valuable insights into the influence of size on the internalization of functional nanocarriers and guidelines for designing MOF-based drug delivery systems for enhanced cancer therapy.

## Data Availability

The data presented in this study are available upon request from the corresponding author.

## Supplementary Materials

The following supporting information can be downloaded at: https://www.mdpi.com/article/10.3390/nano15110826/s1, Figure S1: Scanning electron microscope images of UiO-67 MOFs prepared at 90 °C (a), 120 °C (b), and 150 °C (c) in the absence of PVP; Figure S2: (a) TEM images of PU_40_ MOFs. (b) HAADF-STEM image and corresponding elemental mapping of PU_40_ MOFs; Figure S3: Cytotoxicity of PU_40_ MOFs.
