# Supplementary material for: Optimizing the Size of Zr-Based Metal–Organic Frameworks for Enhanced Anticancer Efficacy
Source: Nanomaterials (Basel). 2025 May 29;15(11):826. doi: 10.3390/nano15110826 (PMC12158041; doi:10.3390/nano15110826)
Supplement: Supplementary file 1 [file nanomaterials-15-00826-s001.zip › nanomaterials-3608895-supplementary.pdf]

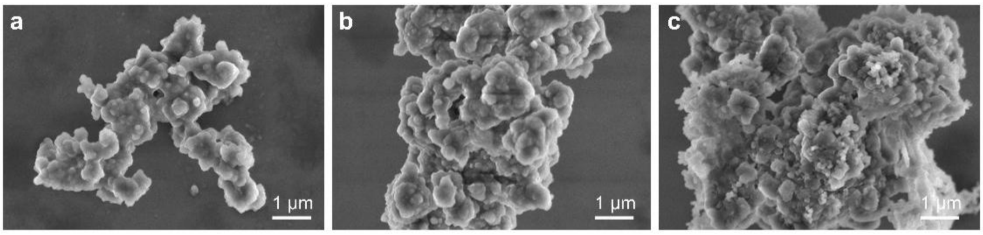

Figure S1. Scanning electron microscope images of UiO-67 MOFs prepared at 90 °C (a), 120 °C (b), and 150 °C (c) in the absence of PVP.

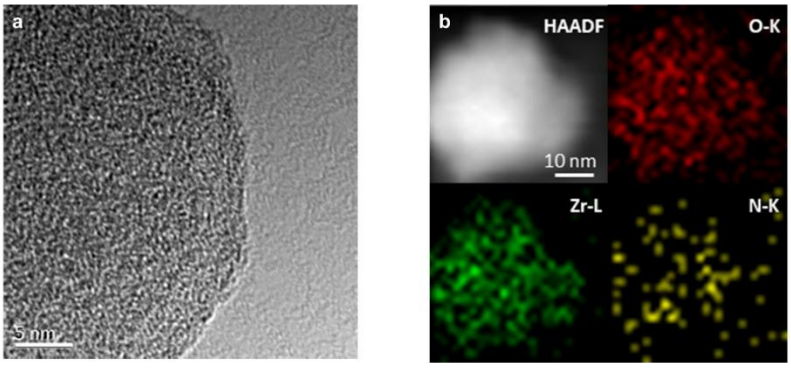

Figure S2. (a) TEM images of PU_{40} MOFs. (b) HAADF-STEM image and corresponding elemental mapping of PU_{40} MOFs.

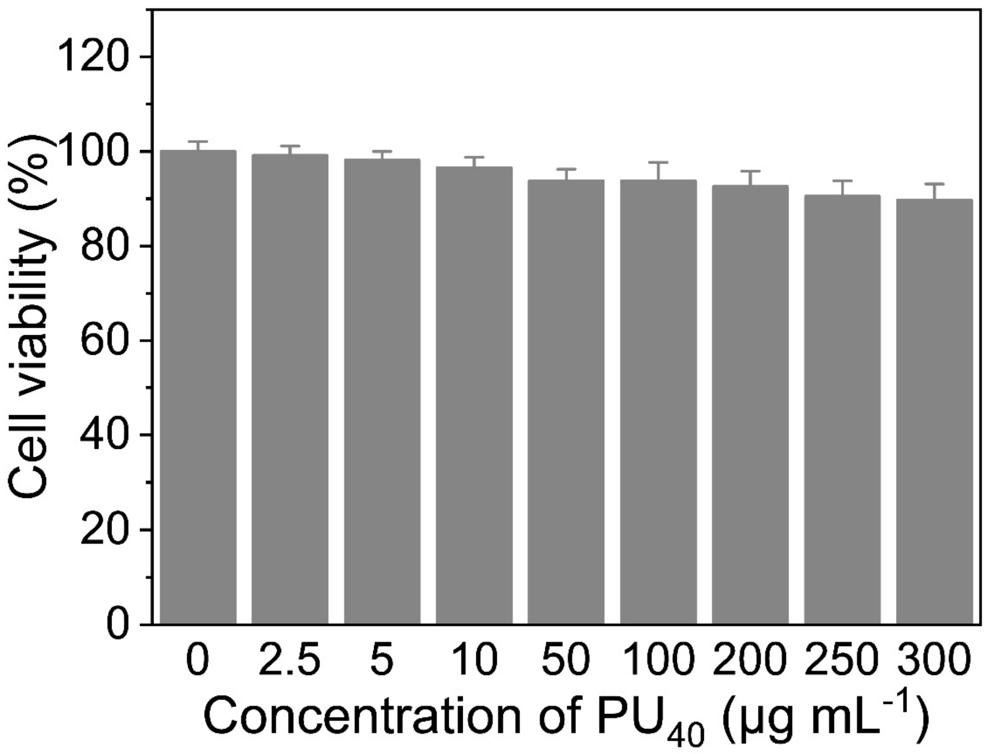

Figure S3. Cytotoxicity of PU_{40} MOFs.
